# Supplementary material for: Nasal Cannula with Long and Narrow Tubing for Non-Invasive Respiratory Support in Preterm Neonates: A Systematic Review and Meta-Analysis
Source: Children (Basel). 2022 Sep 23;9(10):1461. doi: 10.3390/children9101461 (PMC9600105; doi:10.3390/children9101461)
Supplement: Supplementary file 1 [file children-09-01461-s001.zip › children-1919834-supplementary.pdf]

Nasal cannula with long & narrow tubing versus other nasal interfaces for non-invasive respiratory support in preterm neonates: A systematic review and meta-analysis

Table S1: Literature search strategy
Table S2: Statements to communicate the findings of the systematic review
Table S3: Excluded studies
Table S4: Risk of Bias for randomised controlled trials
Table S5: Risk of Bias for non-randomised controlled trials

Table S1: Literature search strategy

Database: Cochrane Library. Search Name: Cannula-ventilation, Date Run:17/01/2021 00:26:44

Search updated on 07-07-2022. Year limited 2021-2022 and 82 new trials downloaded for review update

ID	Search	Hits
#1	MeSH descriptor: [Infant, Premature] explode all trees	3785
#2	MeSH descriptor: [Infant, Low Birth Weight] explode all trees	2191
#3	(Preterm* or premature or "Low birth weight" or lbw or vlbw or elbw	30409
#4	#1 or #3	30409
#5	cannula*	5888
#6	tube OR tubing	17781
#7	CLNT	1
#8	mask	10646
#9	prong*	563
#10	interface	4680
#11	canula	157
#12	RAM NEAR (cannula* or tube*)	21
#13	MeSH descriptor: [Cannula] explode all trees	97
#14	MeSH descriptor: [Masks] explode all trees	1557
#15	{OR #5-#14}	36356
#16	MeSH descriptor: [Intermittent Positive-Pressure Ventilation]	247
#17	MeSH descriptor: [Noninvasive Ventilation] explode all trees	257
#18	Continuous Positive Airway Pressure	4542
#19	NIPPV	359
#20	intermittent positive-pressure ventilation	758
#21	noninvasive ventilation	3688
#22	MeSH descriptor: [Continuous Positive Airway Pressure] explode all	1095
#23	cpap	4864
#24	ventilation	27745
#25	{OR #16-#24}	31846
#26	#4 AND #15 AND #25	833

Database(s): Ovid MEDLINE(R) ALL 1946 to January 14, 2021
Search Strategy:

#	Searches	Results
1	Infant, Premature/	54667
2	Infant, Small for Gestational Age/	7575
3	Infant, Low Birth Weight/	18744
4	Infant, Extremely Premature/	2703
5	Infant, Very Low Birth Weight/	8649

6	(Preterm* or premature or "Low birth weight" or lbw or vlbw or "Low birth weights" or "Low birthweight" or "Low birthweights" or "pre-terms" or "Pre-term" or "Small gestational age" or SGA or "Extremely premature").ab,ti.	215606
7	1 or 2 or 3 or 4 or 5 or 6	236461
8	Tube.mp.	162040
9	Clnt.mp.	7
10	Mask.mp. or Masks/	33922
11	canula*.mp.	709
12	cannula*.mp. [mp=title, abstract, original title, name of substance word, subject heading word, floating sub-heading word, keyword heading word, organism supplementary concept word, protocol supplementary concept word, rare disease supplementary concept word, unique identifier, synonyms]	45825
13	(ram adj3 (cannula* or tube*)).mp. [mp=title, abstract, original title, name of substance word, subject heading word, floating sub-heading word, keyword heading word, organism supplementary concept word, protocol supplementary concept word, rare disease supplementary concept word, unique identifier, synonyms]	28
14	tubing.mp.	7464
15	prong*.mp.	3628
16	8 or 9 or 10 or 11 or 12 or 13 or 14 or 15	248091
17	intermittent positive pressure ventilation.mp. or Intermittent Positive-Pressure Ventilation/	3040
18	Noninvasive Ventilation/ or Continuous Positive Airway Pressure/ or NIPPV.mp.	10045
19	"continuous positive airway pressure".mp.	13013
20	Continuous Positive Airway Pressure/ or cpap.mp.	12275
21	ventilation.mp. or Ventilation/	143094
22	17 or 18 or 19 or 20 or 21	153940
23	7 and 16 and 22	1050
24	limit 23 to clinical trial, all	171

Database(s): **Embase** 1974 to 2021 Week 01
Search Strategy:

#	Searches	Results
1	Infant, Premature/	101009
2	Infant, Small for Gestational Age/	15111
3	Infant, Low Birth Weight/	33240
4	Infant, Extremely Premature/	106004

5	Infant, Very Low Birth Weight/	12669
6	(Preterm* or premature or "Low birth weight" or lbw or vlbw or "Low birth weights" or "Low birthweight" or "Low birthweights" or "pre-terms" or "Pre-term" or "Small gestational age" or SGA or "Extremely premature").ab,ti.	286816
7	1 or 2 or 3 or 4 or 5 or 6	324334
8	Tube.mp.	273799
9	Clnt.mp.	10
10	Mask.mp. or Masks/	66259
11	canula*.mp.	1418
12	cannula*.mp. [mp=title, abstract, heading word, drug trade name, original title, device manufacturer, drug manufacturer, device trade name, keyword, floating subheading word, candidate term word]	75214
13	(ram adj3 (cannula* or tube*)).mp. [mp=title, abstract, heading word, drug trade name, original title, device manufacturer, drug manufacturer, device trade name, keyword, floating subheading word, candidate term word]	56
14	tubing.mp.	10335
15	prong*.mp.	5220
16	8 or 9 or 10 or 11 or 12 or 13 or 14 or 15	417105
17	intermittent positive pressure ventilation.mp. or Intermittent Positive-Pressure Ventilation/	4144
18	Noninvasive Ventilation/ or Continuous Positive Airway Pressure/ or NIPPV.mp.	58808
19	"continuous positive airway pressure".mp.	15359
20	Continuous Positive Airway Pressure/ or cpap.mp.	53996
21	ventilation.mp. or Ventilation/	283057
22	17 or 18 or 19 or 20 or 21	311901
23	7 and 16 and 22	2430
24	limit 23 to embase	1701
25	limit 24 to clinical trial	102

CINAHL: EBSCOhost Research Databases Search Screen - Advanced Search

Monday, January 18, 2021 12:01:15 PM

Search #	Searches	Results
S15	S3 AND S8 AND S13	468
S14	S3 AND S8 AND S13	1,706
S13	S9 OR S10 OR S11 OR S12	42,779
S12	TI (ventilation OR cpap or continuous positive airway pressure or ncpap or nasal continuous positive airway pressure OR NIPPV OR NIV) OR AB (	38,392

	ventilation OR cpap or continuous positive airway pressure or ncpap or nasal continuous positive airway pressure OR NIPPV OR NIV)	
S11	(MH "Continuous Positive Airway Pressure") OR "Continuous Positive Airway Pressure"	6,680
S10	"Noninvasive Ventilation"	1,682
S9	(MH "Intermittent Positive Pressure Ventilation") OR (MH "Positive Pressure Ventilation+") OR (MH "Pressure Support Ventilation") OR (MH "Intermittent Positive Pressure Breathing") OR (MH "Continuous Positive Airway Pressure") OR "Intermittent Positive-Pressure Ventilation"	11,539
S8	S4 OR S5 OR S6 OR S7	66,657
S7	TI (Cannula* OR canula* OR tube OR tubing OR CLNT OR mask* OR prong* OR interface) OR AB (Cannula* OR canula* OR tube OR tubing OR CLNT OR mask* OR prong* OR interface)	65,383
S6	RAM N (cannula OR tube OR canula)	3
S5	(MH "Masks")	2,792
S4	(MH "Nasal Cannula")	558
S3	S1 OR S2	674,257
S2	TI ((Neonate* or Newborn* or Preterm* or term or premature or "Low birth weight" or lbw or vlbw or elbw or "Low birth weights" or "Low birthweight" or "Low birthweights" or Infant* or "pre-terms" or "Pre-term" or "Small gestational age" or SGA or "Extremely premature")) OR AB ((Neonate* or Newborn* or Preterm* or term or premature or "Low birth weight" or lbw or vlbw or elbw or "Low birth weights" or "Low birthweight" or "Low birthweights" or Infant* or "pre-terms" or "Pre-term" or "Small gestational age" or SGA or "Extremely premature"))	510,328
S1	(MH "Infant+") OR (MH "Infant, Premature") OR (MH "Infant, Postmature") OR (MH "Infant, High Risk") OR (MH "Infant, Very Low Birth Weight") OR (MH "Infant, Small for Gestational Age") OR (MH "Infant, Large for Gestational Age") OR (MH "Infant, Newborn, Diseases+")	275,252

Table S2: Statements to communicate the findings of the systematic review

Descriptor	Criteria
Clinical benefit/harm	Statistically significant result<br>High certainty evidence<br>Biological mechanism(s) well established<br>Point estimates of underlying studies are consistently in one direction<br>Optimal information size reached[6]
Probable clinical benefit/harm	Statistically significant result<br>Moderate or high certainty evidence<br>Evidence of biological plausibility<br>Point estimates of underlying studies are predominatly in one direction<br>Close to optimal information size or summary confidence interval is sufficiently narrow to give confidence that the true effect would be clinically meaningful if it is only in the ballpark of the summary estimate.
Possible clinical benefit/harm	Statistically significant result<br>Low or very low certainty evidence<br>Few studies, wide summary confidence interval or effect is driven by one or two heavily weighted studies
Improbable benefit/harm	Statistically non-significant result<br>Moderate or high certainty evidence<br>Point estimates of underlying studies are close to and on both sides of the line of null effect
No clinical benefit/harm	Statistically non-significant result<br>High certainty evidence<br>Point estimates of underlying studies are close to and on either side of the line of null effect<br>Majority of underlying studies are adequately powered for outcome of interest<br>Optimal information size reached reached
Clinical benefit/harm cannot be excluded	Statistically non-significant result<br>Low or very low certainty evidence<br>Few studies<br>Wide confidence intervals
Modified GRADE recommendations (Ref: Santesso N, Glenton C, Dahm P, Garner P, Akl EA, Alper B, Brignardello-Petersen R, Carrasco-Labra A, De Beer H, Hultcrantz M, Kuijpers T, Meerpohl J, Morgan R, et al., GRADE Working Group. GRADE guidelines 26: informative statements to communicate the findings of systematic reviews of interventions. J Clin Epidemiol. 2020 Mar;119:126-135.)	

Table S3: Excluded studies

Study	Reason for exclusion
Green et al (2018) [17]	Compared the resistance of interfaces used to CPAP delivery in neonates, in an in vitro setting.
Gerdes et al ((2014) [19]	Studied the factors influencing delivered mean airway pressure during nasal CPAP with RAM cannula on a simulated test lung model.
Iyer et al (2015) [21]	Measured the transmission of pressures using RAM cannula in an ex vivo experimental design on lung simulator
Bailes et al (2016) [4]	Evaluated the effect of flow and interface type on pressures delivered with Bubble CPAP in a simulated lung model.
Classen et al (2019) [36]	Compared two Bubble CPAP devices Babi-plus and B&B Bubbler using RAM cannula for pressure transmission in Bench studies. In the clinical outcomes, the comparators were the two Bubble CPAP devices.
Matlock et al (2019) [16]	The outcome was tidal volume delivered during NIPPV, which was not of interest for this meta-analysis
Chandrasekharan et al (2017) [37]	Compared the efficacy and safety of continuous positive airway pressure (CPAP) delivered using nasal masks with binasal prongs. The comparator groups did not use RAM cannula as interface and hence were not relevant to this review.
Luistero et al (2020) [38]	Conducted a randomised controlled trial, determined the effectiveness of KMC in neonates on CPAP with RAM cannula. Both the groups received the CPAP through RAM cannula and outcomes were analysed to determine effect of KMC versus no KMC.
Conti et al (2018) [39]	Compared new nasal mask, Fischer Paykel nasal mask and endotracheal tube on infant manikin.

Table S4: Risk of Bias for randomised controlled trials

Study ID	Domain 1. Risk of bias from the randomization process	Domain 2. Risk of bias due to deviations from the intended interventions	Domain 3. Missing outcome data	Domain 4. Risk of bias in measurement of the outcome	Domain 5. Risk of bias in selection of the reported result	Overall Risk of Bias judgement
**Gocke 2019**	Some concerns	Low	Low	Low	Low	Some concerns
**Hoschwald 2021**	Low	Low	Low	Low	Low	Low
**Maram 2019**	Low	Low	Low	Low	Low	Low

Table S5: Risk of Bias for non-randomised controlled trials

Study ID	Bias due to confounding	Bias in selecting participants	Bias in classification of the interventions	Bias due to deviations from intended interventions	Bias due to missing data	Bias in measurement of outcomes	Bias in selection of the reported result	Overall risk of bias
**Sharma 2020**	Serious	Low	Low	Low	Low	Moderate	Low	Serious
**Singh 2018**	Low	Low	Low	Low	Low	Moderate	Low	Moderate
**Drescher 2018**	Serious	Serious	Moderate	Low	Low	Moderate	Moderate	Serious
